# Estimating the associations between personal, health, lifestyle, and social factors on depressive symptoms from adolescence to adulthood: a fixed effect approach using panel data

**DOI:** 10.1093/eurpub/ckag122

**Published:** 2026-08-04

**Authors:** Christine Leonhard Birk Sørensen, Finn B Larsen, Ute Bültmann, Oleguer Plana-Ripoll, Trine N Winding, Pernille B Steen, Karin Biering

**Affiliations:** Department of Occupational & Environmental Medicine, Gødstrup Hospital, Herning, Denmark; DEFACTUM, Central Denmark Region, Aarhus, Denmark; Department of Health Sciences, Community & Occupational Medicine, University Medical Center Groningen, University of Groningen, Groningen, the Netherlands; Department of Clinical Epidemiology, Aarhus University and Aarhus University Hospital, Aarhus, Denmark; National Centre for Register-based Research, Aarhus University, Aarhus, Denmark; Department of Occupational & Environmental Medicine, Gødstrup Hospital, Herning, Denmark; Department of Occupational & Environmental Medicine, Gødstrup Hospital, Herning, Denmark; Department of Occupational & Environmental Medicine, Gødstrup Hospital, Herning, Denmark

## Abstract

Numerous studies have tried to understand the causes of mental health problems, mostly focusing on single exposures. These approaches often fail to capture the complex and interconnected nature of mental health problems. This study aims to investigate the associations of concurrent age-specific changes in explanatory factors, encompassing personal, health, lifestyle, and social factors and depressive symptoms in individuals aged 15 to 32 years. Individuals born in 1989 were followed from 2004 to 2021 with surveys at ages 15, 18, 21, 28, and 32. Inverse probability weights and multiple imputations with chained equations were used to account for attrition and missing data. Descriptive characteristics for each wave were estimated as well as the changes in depressive symptoms and explanatory variables between each wave. Fixed effect regression models and dominance analyses examined the contribution of change in each explanatory variable to the change in depressive symptoms between each wave. Lastly, analyses of asymmetric change were estimated to detect asymmetric associations of explanatory variables and depressive symptoms. The relative importance of the explanatory variables changed between age points. Between all age points, stress was the most dominant variable with a relative contribution above 30% between all age points, while the contribution of sense of coherence increased through the waves from 17% to 25%. Self-esteem, self-rated health, and psychosomatic symptoms had also high dominance with shifting contributions through the ages. The associations between explanatory factors and depressive symptoms are dynamic and preventive strategies should be tailored towards the different life stages.

## Introduction

Mental health problems are rising, affecting individuals across the globe and posing challenges to public health systems [1]. In Denmark, this rise is reflected in increasing numbers of individuals with mental disorder diagnoses, use of psychotropic medications, and prevalence of self-reported mental health problems [2–6]. Numerous studies have tried to understand the underlying causes of mental health problems, often focusing on single exposures, with several systematic reviews synthesizing these findings [7–9]. However, these studies often fail to capture the complex and interconnected nature of mental health.

Recent population-based research has identified a range of potential contributors to increasing mental health problems among adolescents. However, these studies consistently emphasize that these trends are driven by complex, multifaceted changes with no single explanatory factor, and are often limited by a focus on isolated exposures and limited insight into how multiple factors interact and change over time [10–12]. To better understand variation in mental health, it is necessary to move beyond studying isolated factors [13, 14]. Öngür and Paulus recently argued that traditional reductionistic approaches simplify mental health into linear cause-and-effect relationships, neglecting the multifaceted interactions between biological, psychological, social, and environmental factors. The authors suggest that mental health is best understood as complex dynamical systems that evolve through the interplay of multiple influences over time [15]. Addressing mental health effectively demands a shift towards complex systems approaches, integrating multiple variables and accounting for their dynamic nature.

A key aspect of mental health complexity is the timing of exposures and their varying impact over the life course. For example, while social support is a strong protective factor in childhood and adolescence, its influence diminishes in adulthood [7]. This underscores the importance of examining the temporal dynamics of risk and protective factors over time. In particular, the transition from adolescence to adulthood involves major biological, psychological, and social changes affecting depressive symptoms [16].

Previous research has identified a range of factors associated with depressive symptoms, including personal factors such as self-esteem and coping; health-related factors such as stress and psychosomatic symptoms; lifestyle behaviours, including substance use and physical activity; social experiences such as bullying, social relationships, and school-related stress; as well as broader societal conditions [7, 10–12, 17–26]. However, there is a lack of studies examining how changes across multiple domains are simultaneously associated with within-individual changes in depressive symptoms across key developmental transitions from adolescence to adulthood.

Arnett’s theory of emerging adulthood describes this life stage, that does not have the dependency of adolescence nor the responsibility of adulthood, as a distinct period of identity exploration that plays a crucial role in mental health development [27]. Arnett argues that due to demographic shifts, such as delayed marriage, postponed parenthood, and extended education, the uncertainty and instability of this phase now extend to ages 18–25 [27]. Given these unique challenges, studying mental health at multiple time points across adolescence, emerging adulthood, and adulthood is essential for understanding the time changing determinants present in different life stages.

In this study, depressive symptoms are used as a measurable indicator of mental health, capturing variation in subclinical psychological distress within the general population rather than clinical psychiatric disorders. This study aims to investigate the association between concurrent age-specific changes in explanatory factors, encompassing personal, health, lifestyle, and social factors, and changes in depressive symptoms in individuals aged 15–32. By adopting a systems-based perspective and examining within-individual patterns and dynamics of depressive symptoms and related factors from adolescence to adulthood, we seek to provide insights into how preventive strategies can be tailored to different life stages.

## Methods

### Population and setting

The population consists of individuals living in the former Ringkjøbing Amt from the cohort VestLiv. All individuals born in 1989 and living in this area in 2004 were invited to participate (*N* = 3681). The surveys included 3054 individuals (83%) aged 15 in 2004, 2400 (65%) aged 18 in 2007, 2145 (58%) aged 21 in 2010, 2102 (57%) aged 28 in 2017, and 1206 (33%) aged 32 in 2021. In the survey, questions about health, family life, social life, school, work, and well-being were asked. Data from the survey were linked with data from registers by using the individuals’ unique identification numbers from the Danish Civil Registration System [28].

### Variables

#### Depressive symptoms

Depressive symptoms were measured using the four-item version of the Center for Epidemiological Studies Depression Scale for Children (CES-DC4) at ages 15, 18, and 21, while the adult version (CES-D4) was used at ages 28 and 32. The four items of the scales are scored from 0 to 3, resulting in a sum score on 0–12. The higher the score, the more depressive symptoms [29]. The full wording of the questionnaire is available in Fig. S1.

#### Explanatory variables

A number of explanatory variables were selected from the surveys based on the holistic biopsychosocial model [30]. The selection criteria for variables included that the variable must be (i) recognized in the literature as being positively or negatively associated with mental health, (ii) measured consistently across all age points to allow for meaningful comparisons over time, and (iii) modifiable between survey waves, as these are essential targets for interventions aimed at reducing depressive symptoms, whereas fixed characteristics like gender or age may inform risk stratification but are not directly alterable through intervention [31–33]. Since the data collection was not originally designed with the specific method of analysing changes between time points, not all variables in the cohort were applicable for this study. The explanatory variables (see details of origin and scoring in Table S1) were categorized into four groups: personal factors, including coping, self-esteem, and sense of coherence [17–19]; health factors, including stress, self-rated health, and psychosomatic symptoms [20–22]; lifestyle factors, including physical activity, body mass index (BMI), and smoking [23–25]; and social factors, including bullying [26].

#### Characteristics

To describe the characteristics of the individuals in the analytical sample, six register-based covariates (sex, country of origin, mental disorder diagnosis, psychotropic medication use, parental education, and household income) and two variables from the survey at age 15 (subjective social status [SSS] in school and SSS in society) were included (see details about the variables in Supplementary Text 1). Socioeconomic status (SES), a complex but central determinant of mental health [34, 35], was represented using four indicators to reflect multiple dimensions: parental education, household income, SSS in school, and SSS in society.

### Statistical methods

#### Participation and missing data

The analytical sample included people who responded to depressive symptom questions in at least three waves, as fewer responses led to excessive missing data. To account for selection bias due to non-participation, inverse probability weights (IPWs) were used [36]. The probability of being in the analytical sample was calculated by sex, mental disorder diagnoses, medication use, country of origin, mean depressive symptoms across surveys, parental mental disorder diagnoses, and several SES measures (parental education, own education, own labour market participation, and equalized household income). The covariates in the model were chosen based on a drawing of Directed Acyclic Graphs (Fig. S1).

To handle data in the analytical sample, multiple imputations (MIs) with chained equations were used on explanatory variables and depressive symptoms in all surveys. Information across the surveys was used in the models together with register data on sex, SES, medication use, and mental disorder diagnosis [37]. After imputation, the size of the population was the same across surveys.

A dropout analysis was conducted to evaluate if the analytical sample differed from the excluded sample, consisting of people answering the question about depressive symptoms in less than three survey waves.

#### Descriptive statistics

The characteristics of the analytical sample were described with counts and percentages for all levels of the categorical characteristics. Moreover, the depressive symptoms according to each of the characteristics were described for each age and the mean changes in depressive symptoms between age points. As the aim of the study was to examine age-specific changes, all analyses of change were done between the five timpoints separately: ages 15–18, ages 18–21, ages 21–28, and 28–32.

The mean and prevalence of the explanatory variables and the within-person changes in the variables between the age points were described. As some change in the variable needed to be present to evaluate if changes in an explanatory factor were associated with changes in depressive symptoms, it was examined if any variables had less than 10% change between the age points to determine whether the variable should be included [38].

#### Analyses

Fixed effects (FEs) regression models were used to examine the association between changes in explanatory variables and changes in depressive symptoms between each age point from ages 15 to 32. Changes were calculated over the same intervals, and the analyses estimated associations between concurrent within-individual changes rather than temporally ordered or causal effects. The depressive symptoms score was treated as the dependent variable, with explanatory variables included as independent variables in models of with-in change. The estimates of the FE regression were compared with estimates of a pooled ordinary least squares (POLS) model using the Hausman test to confirm which models were the best fit for the panel data [38]. As the FE regression is based on within-individual change, all time-invariant confounding was adjusted for in the models. All explanatory variables were included in the models and thereby mutually accounted for. Furthermore, the models were adjusted for the survey wave to control for changes in the outcome and explanatory variables due to aging of the cohort and time trends [38, 39]. Dominance analyses were used to examine the relative importance of the explanatory variables. The method decomposes and compares the contribution to the explained variance in the model of each independent variable by applying the dominance analysis to the within variance of the FE regression. Thereby, the analyses show how much of the intra-individual variance in the depressive symptoms that was explained by the change in the single variables [40, 41].

Analyses were conducted to examine if the association between any of the explanatory variables and depressive symptoms was asymmetrical. A modified version of the first-difference method, developed by Paul D. Allison [39], was used. The first difference method was used in both the FE and the asymmetrical change analyses [38, 39].

## Results

The descriptive analyses showed that the mean depressive symptoms were highest at age 18 (2.85; 95% confidence interval [CI]: 2.73–2.97) and the mean absolute changes in depressive symptoms between all age points ranged between 1.64 and 2.04 (Table 1). Females generally had higher mean depressive symptoms than males and showed larger mean absolute changes over time. Individuals of non-Danish origin had higher mean depressive symptoms at all age points except age 15. Different patterns were observed across SES measures. Individuals with low household income, low SSS in society, and low SSS in school generally reported higher mean depressive symptoms compared to those individuals in the middle and high SES groups. However, depressive symptoms were of similar magnitude between individuals with short and long parental education levels at several age points. Finally, individuals who received a mental disorder diagnosis or used psychotropic medication during the study period—including after the ages at which different surveys were responded to—had higher mean depressive symptoms and greater absolute changes in depressive symptoms at all age points compared to those individuals without a diagnosis or medication use.

**Table 1.** Characteristics of the analytical sample at baseline (2004) with mean depressive symptoms scores (CES-D(C)4) and absolute change between time points

		Depressive symptoms (mean [95% CI])					Absolute change in depressive symptoms (Mean [95% CI])			
	*N* (%)	15	18	21	28	32	15–18	18–21	21–28	28–32
Total	2157 (100%)	2.23 (2.12–2.33)	2.85 (2.73–2.97)	2.50 (2.37–2.62)	2.65 (2.54–2.76)	2.69 (2.55–2.83)	2.04 (1.95–2.14)	1.95 (1.85–2.04)	1.87 (1.78–1.96)	1.64 (1.55–1.74)
Sex										
Males	1122 (52%)	1.89 (1.74–2.04)	2.44 (2.28–2.61)	2.23 (2.05–2.40)	2.58 (2.40–2.76)	2.63 (2.42–2.83)	1.83 (1.70–1.96)	1.75 (1.62–1.89)	1.80 (1.67–1.93)	1.64 (1.50–1.78)
Females	1035 (48%)	2.59 (2.44–2.74)	3.29 (3.14–3.44)	2.79 (2.63–2.94)	2.72 (2.58–2.86)	2.76 (2.58–2.94)	2.28 (2.15–2.40)	2.15 (2.03–2.27)	1.95 (1.84–2.06)	1.65 (1.53–1.76)
Country of origin										
Denmark	2035 (94.4%)	2.24 (2.13–2.34)	2.84 (2.72–2.95)	2.48 (2.36–2.60)	2.63 (2.52–2.74)	2.65 (2.52–2.79)	2.05 (1.96–2.14)	1.95 (1.86–2.05)	1.86 (1.78–1.95)	1.62 (1.53–1.72)
Other	122 (5.6%)	2.01 (1.26–2.76)	3.05 (2.35–3.75)	2.74 (2.08–3.40)	2.91 (2.23–3.59)	3.33 (2.61–4.06)	1.96 (1.33–2.58)	1.81 (1.24–2.38)	2.02 (1.50–2.54)	1.99 (1.45–2.52)
Parental education										
Long	128 (6%)	2.29 (1.94–2.64)	3.27 (2.83–3.70)	2.49 (2.10–2.89)	2.93 (2.56–3.30)	2.89 (2.46–3.33)	2.15 (1.83–2.47)	2.23 (1.89–2.57)	1.92 (1.62–2.21)	1.53 (1.23–1.82)
Middle	1670 (77%)	2.21 (2.10–2.32)	2.79 (2.67–2.91)	2.46 (2.33–2.58)	2.58 (2.47–2.69)	2.62 (2.47–2.76)	2.00 (1.90–2.10)	1.92 (1.82–2.01)	1.85 (1.76–1.94)	1.63 (1.53–1.72)
Short	359 (17%)	2.28 (1.92–2.64)	2.98 (2.60–3.35)	2.67 (2.28–3.06)	2.87 (2.48–3.26)	2.96 (2.54–3.38)	2.22 (1.91–2.52)	1.97 (1.66–2.29)	1.96 (1.68–2.24)	1.78 (1.50–2.05)
Household income										
High	413 (19%)	2.02 (1.82–2.22)	2.86 (2.63–3.09)	2.19 (1.96–2.42)	2.50 (2.29–2.70)	2.46 (2.22–2.71)	2.10 (1.91–2.30)	1.85 (1.67–2.02)	1.73 (1.56–1.89)	1.54 (1.38–1.70)
Middle	1333 (62%)	2.25 (2.11–2.38)	2.79 (2.65–2.92)	2.45 (2.31–2.59)	2.58 (2.44–2.72)	2.64 (2.49–2.79)	2.04 (1.93–2.15)	1.98 (1.87–2.10)	1.88 (1.77–1.98)	1.63 (1.52–1.75)
Low	411 (19%)	2.37 (2.06–2.68)	3.05 (2.72–3.38)	2.96 (2.61–3.31)	3.03 (2.71–3.34)	3.09 (2.73–3.46)	2.00 (1.74–2.25)	1.92 (1.68–2.16)	1.99 (1.75–2.23)	1.78 (1.56–2.00)
SSS in society										
High	107 (5%)	4.72 (3.83–5.62)	4.60 (3.85–5.35)	4.15 (3.41–4.88)	3.56 (2.92–4.20)	3.78 (3.12–4.44)	2.78 (2.22–3.33)	2.27 (1.73–2.80)	2.35 (1.88–2.82)	1.96 (1.51–2.42)
Middle	1142 (53%)	2.27 (2.13–2.41)	2.96 (2.80–3.13)	2.58 (2.41–2.74)	2.78 (2.61–2.94)	2.80 (2.61–2.99)	2.12 (1.98–2.25)	1.99 (1.85–2.12)	1.90 (1.78–2.01)	1.69 (1.57–1.81)
Low	908 (42%)	1.88 (1.73–2.02)	2.50 (2.35–2.66)	2.20 (2.04–2.36)	2.37 (2.22–2.53)	2.42 (2.23–2.61)	1.87 (1.74–2.00)	1.86 (1.72–1.99)	1.78 (1.65–1.90)	1.55 (1.42–1.68)
SSS in school										
High	46 (2%)	3.76 (2.85–4.67)	3.59 (2.56–4.62)	3.93 (2.66–5.20)	3.88 (2.59–5.18)	3.53 (2.42–4.63)	2.13 (1.46–2.81)	2.15 (1.37–2.94)	2.17 (1.49–2.84)	1.77 (0.94–2.61)
Middle	1469 (68%)	2.30 (2.16–2.43)	2.95 (2.80–3.10)	2.60 (2.45–2.75)	2.67 (2.53–2.81)	2.77 (2.61–2.94)	2.10 (1.98–2.21)	1.97 (1.85–2.09)	1.90 (1.79–2.00)	1.68 (1.57–1.79)
Low	642 (30%)	1.96 (1.77–2.15)	2.57 (2.38–2.75)	2.15 (1.96–2.34)	2.50 (2.31–2.69)	2.44 (2.23–2.65)	1.92 (1.77–2.07)	1.88 (1.73–2.03)	1.79 (1.64–1.93)	1.55 (1.42–1.69)
Mental disorder diagnosis										
Older child (5–12 years)	64 (3%)	2.24 (1.42–3.07)	2.51 (1.68–3.35)	2.68 (1.84–3.52)	3.20 (2.26–4.15)	2.70 (1.71–3.69)	1.82 (1.18–2.45)	1.78 (1.15–2.41)	1.86 (1.20–2.51)	1.53 (0.91–2.16)
Adolescent (13–17 years)	106 (5%)	3.08 (2.38–3.79)	4.19 (3.48–4.90)	3.47 (2.71–4.23)	3.59 (2.84–4.34)	3.00 (2.14–3.86)	2.94 (2.36–3.52)	2.19 (1.65–2.73)	1.98 (1.50–2.46)	1.98 (1.45–2.52)
Adult (18–32 years)	373 (17%)	2.88 (2.55–3.21)	3.60 (3.25–3.95)	3.53 (3.14–3.91)	3.67 (3.35–3.99)	3.85 (3.45–4.25)	2.51 (2.25–2.78)	2.15 (1.88–2.42)	2.15 (1.92–2.38)	1.95 (1.72–2.18)
Study period (5–32 years)	451 (21%)	2.76 (2.46–3.06)	3.51 (3.18–3.83)	3.32 (2.98–3.67)	3.42 (3.12–3.72)	3.48 (3.10–3.85)	2.49 (2.24–2.75)	2.13 (1.88–2.38)	2.05 (1.83–2.27)	1.86 (1.65–2.08)
None in study period	1706 (79%)	2.09 (1.97–2.20)	2.68 (2.56–2.79)	2.28 (2.16–2.39)	2.44 (2.33–2.56)	2.48 (2.35–2.62)	1.93 (1.83–2.02)	1.90 (1.80–1.99)	1.82 (1.73–1.92)	1.59 (1.48–1.69)
Medication use										
Older child (5–12 years)	22 (1%)	1.81 (0.64–2.98)	2.88 (0.82–4.94)	3.56 (1.25–5.87)	2.65 (0.90–4.40)	3.13 (0.70–5.55)	2.22 (0.56–3.88)	1.94 (0.75–3.13)	2.39 (1.13–3.64)	1.67 (0.50–2.84)
Adolescent (13–17 years)	66 (3%)	3.24 (2.46–4.02)	3.87 (2.99–4.76)	3.61 (2.70–4.51)	3.09 (2.26–3.92)	3.04 (2.06–4.01)	2.64 (2.03–3.25)	2.69 (2.15–3.22)	2.33 (1.83–2.83)	1.73 (1.21–2.25)
Adult (18–32 years)	601 (28%)	2.80 (2.54–3.05)	3.49 (3.21–3.76)	3.38 (3.09–3.67)	3.56 (3.30–3.81)	3.54 (3.24–3.84)	2.41 (2.22–2.59)	2.27 (2.08–2.46)	2.16 (1.99–2.33)	1.86 (1.69–2.03)
Study period (5–32 years)	667 (31%)	2.71 (2.47–2.95)	3.40 (3.15–3.66)	3.27 (3.00–3.55)	3.43 (3.19–3.67)	3.41 (3.13–3.70)	2.36 (2.18–2.53)	2.26 (2.08–2.43)	2.15 (1.99–2.31)	1.83 (1.67–2.00)
None in study period	1490 (69%)	2.01 (1.90–2.12)	2.60 (2.49–2.72)	2.15 (2.03–2.26)	2.30 (2.18–2.41)	2.37 (2.22–2.51)	1.92 (1.82–2.03)	1.83 (1.73–1.93)	1.74 (1.65–1.84)	1.55 (1.45–1.65)

SSS: subjective social status.

The dropout analyses showed that the excluded sample, compared to the analytical sample, included more males, individuals born outside Denmark, lower levels of parental education, lower household income, more mental disorder diagnoses, and greater use of psychotropic medication (Table S2). IPWs reduced these differences across all variables but did not fully eliminate differences between the excluded and analytical sample for sex, origin, parental education, household income, mental disorder diagnoses in adulthood and during the study period, or psychotropic medication use in adulthood and during the study period.

Descriptive analyses of changes in explanatory variables showed that the distribution of categorical variables and the mean of continuous variables differed between age points (Table 2). All variables had above 10% change between age points and therefore no variables had to be reconsidered for inclusion (Table S3). The variables changing the least between age points were smoking and bullying, while the variables changing the most were coping, stress, and BMI.

**Table 2.** Mean and prevalences of explanatory variables

	Mean (95% CI)/*N* (%)				
	Age 15	Age 18	Age 21	Age 28	Age 32
Personal factors					
Coping	16.90 (16.72–17.08)	19.11 (18.92–19.30)	15.60 (15.40–15.79)	15.41 (15.21–15.62)	15.48 (15.21–15.74)
Self-esteem	11.03 (10.89–11.17)	10.43 (10.28–10.58)	10.03 (9.86–10.20)	10.04 (9.87–10.22)	10.16 (9.96–10.36)
Sense of coherence	9.75 (9.65–9.86)	9.82 (9.70–9.94)	9.67 (9.54–9.81)	8.85 (8.70–9.00)	9.05 (8.88–9.21)
Health factors					
Stress	9.23 (9.11–9.36)	9.14 (9.00–9.28)	9.47 (9.31–9.63)	8.90 (8.75–9.06)	9.08 (8.89–9.27)
Psychosomatic symptoms	3.07 (2.94–3.20)	3.23 (3.10–3.36)	3.10 (2.96–3.25)	3.43 (3.29–3.57)	3.81 (3.69–3.94)
Self-rated health					
Excellent	626 (29%)	472 (22%)	670 (31%)	421 (20%)	372 (17%)
Very good	984 (46%)	860 (40%)	897 (42%)	941 (44%)	946 (44%)
Good	460 (21%)	645 (30%)	449 (21%)	586 (27%)	586 (27%)
Fair	80 (4%)	152 (7%)	123 (6%)	183 (9%)	214 (10%)
Poor	8 (0%)	29 (1%)	18 (1%)	27 (1%)	39 (2%)
Lifestyle factors					
Physical activity					
7+ hours	479 (22%)	328 (15%)	311 (14%)	236 (11%)	164 (8%)
4–6 hours	725 (34%)	616 (29%)	556 (26%)	490 (23%)	368 (17%)
2–3 hours	588 (27%)	657 (30%)	655 (30%)	674 (31%)	707 (33%)
1 hour	234 (11%)	319 (15%)	278 (13%)	318 (15%)	370 (17%)
1/2 hour	88 (4%)	126 (6%)	188 (9%)	205 (10%)	288 (13%)
None	43 (2%)	111 (5%)	169 (8%)	234 (11%)	259 (12%)
Smoking					
No	1889 (88%)	1536 (71%)	1449 (67%)	1600 (74%)	1727 (80%)
Rarely	96 (4%)	152 (7%)	139 (6%)	114 (5%)	89 (4%)
Weekly	45 (2%)	92 (4%)	117 (5%)	443 (21%)	77 (4%)
Daily	127 (6%)	378 (18%)	452 (21%)	0 (0%)	265 (12%)
BMI	20.29 (20.14–20.45)	22.53 (22.34–22.72)	23.80 (23.58–24.02)	25.55 (25.27–25.84)	26.30 (25.98–26.63)
Social factors					
Bullying					
Never	1567 (73%)	1792 (83%)	1964 (91%)	1998 (93%)	1960 (91%)
Once or twice	378 (18%)	267 (12%)	144 (7%)	94 (4%)	89 (4%)
Sometimes	153 (7%)	73 (3%)	21 (1%)	35 (2%)	56 (3%)
Weekly	31 (1%)	15 (1%)	22 (1%)	31 (1%)	52 (2%)
Several times a week	27 (1%)	9 (0%)	6 (0%)	0 (0%)	0 (0%)

**Figure 1.**
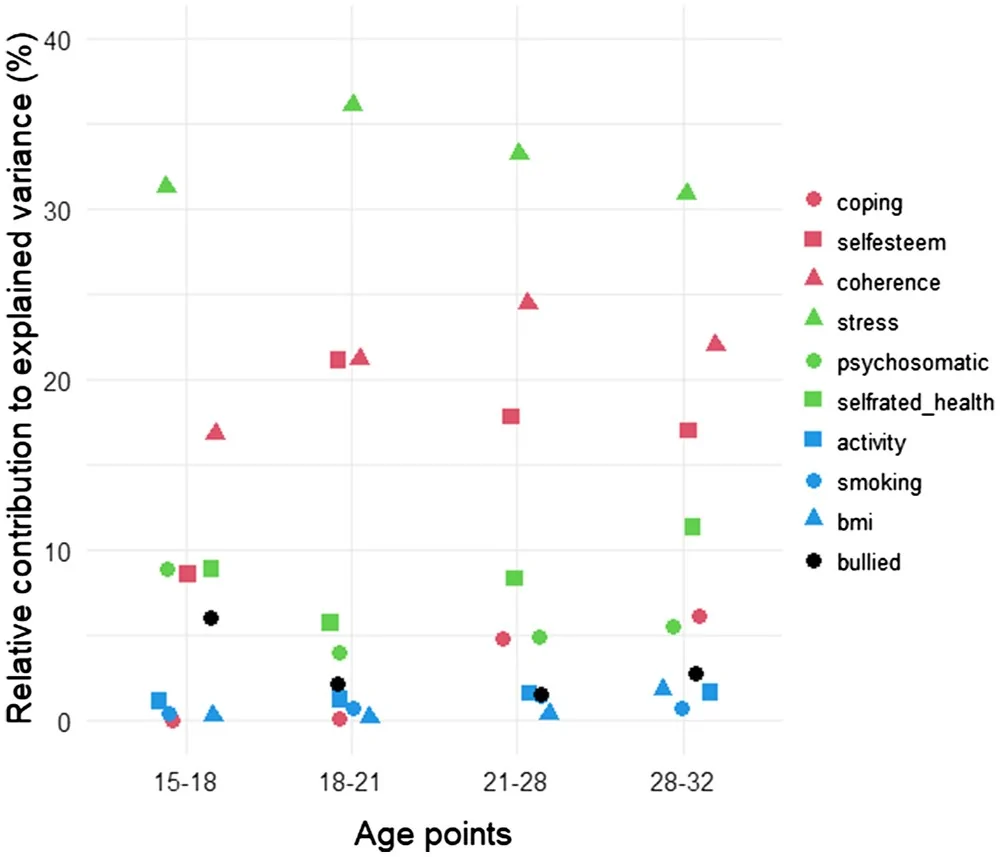
Results from the dominance analyses showing the relative contribution to the explained variance of depressive symptoms for personal (red), health (green), lifestyle (blue), and social factors (black).

The Hausman test found the FE model to be better fitting than POLS between all age points (Table S4). Results from the FE analyses showed that the models explained between 0.25 and 0.29 of the total variance in depressive symptoms and that changes in most explanatory variables were associated with changes in depressive symptoms (Table S5). For example, one unit change in stress between ages 15 and 18 was associated with a 0.25 (0.19–0.30) change in depressive symptoms in the same period. The dominance analyses showed that the variable that contributed the most to the change in depressive symptoms between all age points was stress, while sense of coherence contributed second most between all age points (Fig. 1). The highest relative contribution to the explained variance for stress was at ages 18–21 (36.13) and the highest relative contribution for sense of coherence was at ages 21–28 (24.51). The variables that contributed the least to change in depressive symptoms were coping between ages 15–18 and 18–21, BMI between ages 21 and 28, and smoking between ages 28 and 32. Self-esteem contributed third most to the explained variance between all age points except at ages 15–18, where self-rated health and psychosomatic symptoms contributed more. The size of the dominance differed between age points. For example, self-rated health had a relative importance above 8% between all age points except at ages 18–21 and sense of coherence had a relative importance of above 21% between all age points but not at ages 15–18.

The analyses of asymmetrical changes showed that psychosomatic symptoms had asymmetric associations at ages 15–18 (*P* = .03; Table 3). A reduction of one score in psychosomatic symptoms between the ages 15 and 18 was associated with a reduction of 0.08 (95% CI: −0.18–0.01) in depressive symptoms, while an increase of one score in psychosomatic symptoms was associated with an increase of 0.24 (95% CI: 0.15–0.33) in depressive symptoms. An indication of asymmetry was found in the association of bullying between ages 28 and 32. However, the wide CIs indicated considerable uncertainty around these estimates, making it difficult to draw firm conclusions about the asymmetrical effect of bullying (*P* = .35).

**Table 3.** Results from asymmetrical change analyses

	Change in depressive symptoms (mean [95% CI])			
	Ages 15–18	Ages 18–21	Ages 21–28	Ages 28–32
Personal factors				
Coping	*P* = .16	*P* = .59	*P* = .59	*P* = .53
1 positive change	−0.02 (−0.05; 0.01)	0.01 (−0.04; 0.06)	0.03 (−0.02; 0.09)	0.03 (−0.02; 0.09)
1 negative change	−0.03 (−0.08; 0.02)	0.00 (−0.02; 0.03)	−0.01 (−0.06; 0.04)	−0.01 (−0.06; 0.04)
Self-esteem	*P* = .49	*P* = .15	*P* = .69	*P* = .55
1 positive change	0.09 (0.01; 0.16)	0.08 (0.01; 0.16)	0.10 (0.03; 0.16)	0.09 (0.02; 0.15)
1 negative change	−0.04 (−0.11; 0.03)	−0.18 (−0.25; −0.12)	−0.11 (−0.18; −0.04)	−0.07 (−0.13; −0.01)
Sense of coherence	*P* = .15	*P* = .67	*P* = .16	*P* = .36
1 positive change	0.21 (0.13; 0.29)	0.17 (0.08; 0.25)	0.11 (−0.00; 0.21)	0.09 (−0.01; 0.19)
1 negative change	−0.10 (−0.19; −0.02)	−0.15 (−0.23; −0.07)	−0.22 (−0.29; −0.15)	−0.16 (−0.23; −0.09)
Health factors				
Stress	*P* = .18	*P* = .31	*P* = .62	*P* = .56
1 positive change	0.20 (0.11; 0.28)	0.18 (0.12; 0.25)	0.21 (0.13; 0.28)	0.20 (0.13; 0.26)
1 negative change	−0.30 (−0.38; −0.22)	−0.25 (−0.34; −0.17)	−0.17 (−0.26; −0.09)	−0.20 (−0.26; −0.15)
Psychosomatic symptoms	*P* = .03	*P* = .63	*P* = .44	*P* = .54
1 positive change	0.24 (0.15; 0.33)	0.09 (0.00; 0.18)	0.09 (−0.00; 0.17)	0.11 (0.01; 0.22)
1 negative change	−0.08 (−0.18; 0.01)	−0.08 (−0.17; 0.01)	−0.03 (−0.13; 0.07)	−0.11 (−0.20; −0.03)
Self-rated health	*P* = .55	*P* = .65	*P* = .62	*P* = .58
1 positive change	0.13 (−0.06; 0.32)	0.12 (−0.14; 0.38)	0.26 (0.08; 0.44)	0.26 (0.10; 0.42)
1 negative change	−0.25 (−0.54; 0.03)	−0.16 (−0.33; 0.01)	−0.24 (−0.47; −0.01)	−0.29 (−0.51; −0.07)
Lifestyle factors				
Physical activity	*P* = .18	*P* = .45	*P* = .62	*P* = .56
1 positive change	−0.09 (−0.22; 0.04)	0.09 (−0.03; 0.21)	0.02 (−0.13; 0.18)	0.03 (−0.07; 0.13)
1 negative change	−0.10 (−0.30; 0.09)	−0.00 (−0.15; 0.14)	0.03 (−0.11; 0.17)	0.01 (−0.13; 0.14)
Smoking	*P* = .68	*P* = .34	*P* = .35	*P* = .51
1 positive change	−0.06 (−0.19; 0.08)	−0.04 (−0.20; 0.12)	0.12 (−0.08; 0.32)	−0.07 (−0.32; 0.18)
1 negative change	0.02 (−0.39; 0.43)	0.20 (−0.02; 0.41)	0.01 (−0.12; 0.14)	0.11 (−0.00; 0.22)
BMI	*P* = .43	*P* = .45	*P* = .59	*P* = .57
1 positive change	0.01 (−0.05; 0.07)	0.03 (−0.03; 0.10)	−0.03 (−0.08; 0.01)	−0.03 (−0.06; 0.00)
1 negative change	−0.09 (−0.27; 0.08)	0.04 (−0.11; 0.19)	0.01 (−0.06; 0.09)	0.03 (−0.04; 0.09)
Social factors				
Bullying	*P* = .73	*P* = .33	*P* = .51	*P* = .35
1 positive change	0.29 (−0.03; 0.60)	−0.14 (−0.52; 0.24)	−0.03 (−0.37; 0.32)	0.22 (−0.01; 0.45)
1 negative change	−0.31 (−0.52; −0.11)	−0.17 (−0.51; 0.17)	0.03 (−0.26; 0.33)	−0.03 (−0.31; 0.25)

The largest negative and positive associations with depressive symptoms were observed in stress, bullying, self-esteem, sense of coherence, and self-rated health with different sizes between the age points. These variables, with exception of bullying, were also the variables with the highest dominance (Fig. 1). Between ages 15 and 18, improvements in bullying and stress were associated with the largest reductions in depressive symptoms, while worsening of bullying and psychosomatic symptoms was associated with the largest increases. Between ages 18 and 21, improvements in self-esteem and stress showed the largest reductions in depressive symptoms, whereas worsening of stress and sense of coherence showed the largest increases. Between ages 21 and 28, improvements in self-rated health and sense of coherence showed the largest reductions in depressive symptoms, while worsening of self-rated health showed the largest increase. Lastly, between ages 28 and 31, improvements in self-rated health and stress were associated with the largest reduction depressive symptoms the most, while worsening of self-rated health and bullying was associated with the largest increases.

## Discussion

This study found that changes in personal, health, lifestyle, and social factors were associated with changes in depressive symptoms among individuals aged 15–32 and that the relative importance of these factors varied across age points. In general, personal and health factors showed the strongest associations with depressive symptoms. These findings emphasize the developmental shifts in the factors influencing depressive symptoms, suggesting that targeted prevention strategies should be tailored to specific life stages.

To our knowledge, this is the first study to apply an FE approach to examine the patterns and dynamic relationships between a range of explanatory factors and depressive symptoms across adolescence and adulthood. We identified stress, sense of coherence, self-esteem, self-rated health, and psychosomatic symptoms as the most dominant variables across five age points. All these factors have previously been found to be associated with mental health in studies focusing on individual risk factors [17–22]. Importantly, the ranking and effect sizes of these variables shifted over time. While stress was the most dominant and sense of coherence the second most dominant factor through all waves, the size of the dominance changed. Self-esteem ranked third in the latest age points (ages 28–32) but only fifth during adolescence (ages 15–18). Low self-esteem, particularly when combined with identity challenges, has been shown to negatively affect mental health [18]. This is consistent with the theory of emerging adulthood (ages 18–25) as a period of identity formation [27]. However, the high dominance of self-esteem at ages 28–32 is less often discussed and warrants further exploration. Self-rated health ranked third at ages 15–18 and fourth in later ages. While longitudinal studies—primarily in much older populations—have shown mixed findings, comparable research in younger age groups is lacking, making direct comparisons difficult [22]. Our study provides insight into this relationship during earlier life stages. Psychosomatic symptoms ranked fourth at ages 15–18 and fifth in later ages, suggesting a slight decline in associations. Previous literature has primarily focused on psychosomatic symptoms in adolescence as predictors of early adult mental health [21]. Bullying did not show high overall dominance but had a strong association with depressive symptoms between ages 15 and 18 (Table 3). Bullying is known to have serious and potentially long-lasting consequences for mental health in children and adolescents, but the temporal impact across developmental stages remains underexplored [26]. This study emphasizes that even though bullying is a rare phenomenon, when occurring, it has severe consequences.

While more variables could have been relevant in a systems-based approach on depressive symptoms, we selected factors representing four broad domains: personal, health, lifestyle, and social. Unfortunately, no consistently measured variables related to social connections, social support, or loneliness were available across all surveys, which likely contributed to the models explaining only 25%–29% of the total variance. Future research should aim to include a broader range of social determinants to improve explanatory power.

While this study contributes to a better understanding of the patterns and dominance over time, future research should explore the potential mediators and interactions between variables. For example, individuals with high self-esteem may be more likely to use active coping strategies in response to stress, thereby protecting their mental health. Similarly, coping strategies are known to buffer the impact of stress [42]. A deeper understanding of these mechanisms would provide valuable insights into the complexity of depressive symptoms over time.

Because changes in explanatory variables and depressive symptoms were measured concurrently, we cannot infer directionality. It remains unclear whether a change in an explanatory variable caused a change in depressive symptoms or vice versa. Therefore, no causal conclusions based on these data and methods can be drawn and we encourage future research into the causal relationships.

This study highlights the importance of age-specific prevention strategies to reduce depressive symptoms in young people. Across all ages, stress and sense of coherence consistently emerged as the most influential factors. While the present study does not provide evidence on specific intervention strategies, these findings highlight the potential value of approaches that address depressive symptoms and related factors such as stress regulation, coping resources, and resilience across multiple domains. However, the results also suggest that the prominence of other factors may vary depending on the developmental stage. For adolescents aged 15–18, reducing stress and bullying may yield the greatest benefit, while among individuals aged 18–21, strategies aimed at improving self-esteem—alongside continued efforts to reduce stress and bullying—appear most relevant. In later ages, ages 21–28 and ages 28–31, improvements in sense of coherence and self-rated health seem especially important. It is worth noting, however, that the minimal detectable change for the four-item CES-DC among 15- to 16-year-olds has been estimated at 3.85 points (95% CI: 2.91; 4.80), suggesting that changes in a single factor may not be sufficient to produce a clinically meaningful reduction in depressive symptoms [43]. Furthermore, the mean levels of depressive symptoms were relatively low across all age points (approximately 2.5 on a scale from 0 to 12), indicating that the observed changes reflect variation within a generally low symptom range. Therefore, preventive efforts should take a multidimensional approach that targets multiple, interacting factors simultaneously, rather than focusing on single factors in isolation. Importantly, some of the explanatory variables included in this study, such as self-esteem, coping, and aspects of lifestyle, may conceptually overlap with depressive symptoms, reflecting related dimensions of broader psychological functioning rather than independent risk factors. Because depressive symptoms may both influence and be influenced by factors such as self-esteem, stress, and sense of coherence, these findings underscore the importance of approaches that address a broader constellation of interrelated psychological and social factors.

One key strength of this study is the use of FE models, which control for all time-invariant confounders, thereby enhancing the internal validity of the results. The design spanning multiple age points—from adolescence through adulthood—provides a unique opportunity to explore changes in depressive symptoms across key developmental transitions. Furthermore, the combination of self-reported and register-based data adds breadth to the analysis. The study also employed MI and IPW to handle missing data and reduce potential bias due to attrition, which helps to strengthen the generalizability of the findings. In addition, the inclusion of a broad set of explanatory variables allows for a more comprehensive examination of the complex interplay between personal, health, lifestyle, and social factors in relation to mental health outcomes.

Nevertheless, several limitations must be considered. The time intervals between age points varied, with changes assessed over 3 years in the first two intervals, 7 years in the third, and 4 years in the final interval. This variation complicates comparisons across models, as the magnitude of change may differ simply due to the length of the follow-up. Moreover, not all variables were measured identically across waves. For example, one item in the measure of sense of coherence was slightly reworded at age 15 to ensure age-appropriate comprehension. While necessary from a developmental standpoint, such changes may affect measurement consistency. Despite efforts to reduce bias using IPW, differences between the analytical sample and the excluded sample persisted on several characteristics, indicating a continued risk of selection bias. Finally, the models explained only 25%–29% of the total variance in depressive symptoms, suggesting that additional unmeasured factors likely contribute to these changes and should be investigated in future research.

## Conclusion

Our findings underscore the importance of considering age-specific changes when designing preventive efforts for depressive symptoms in young people. While changes in personal- and health-related factors had the strongest associations with changes in depressive symptoms, their relative importance varied across different age points. Bullying in adolescence also played a role in depressive symptoms, but our ability to fully capture the impact of social factors was limited by the available data. Future research should further explore the role of social determinants in depressive symptoms using a system-based approach to better inform targeted interventions across different life stages.

## Supplementary Material

ckag122_Supplementary_Data

## Data Availability

Data cannot be shared, as they have been made available for the first author specifically via Statistics Denmark. On reasonable request, the codes are available from the corresponding author.

## Notes

Further references cited in Supplementary file.
