# Supplementary material for: Estimating the associations between personal, health, lifestyle, and social factors on depressive symptoms from adolescence to adulthood: a fixed effect approach using panel data
Source: Eur J Public Health. 2026 Aug 4;36(5):ckag122. doi: 10.1093/eurpub/ckag122 (PMC13437016; doi:10.1093/eurpub/ckag122)
Supplement: Supplementary file 1 [file ckag122_supplementary_data.zip › ejph-2025-05-om-0371-File007.docx]

| **Supplementary Table 1:** Scoring and definition of the explanatory variables, including differences in measurement over time | |
| --- | --- |
| **Explanatory Variable** | |
| **Personal factors** | |
| Coping | 7 items from “Brief COPE scale” each scored 1-4 (7-28 total score) (24). Questions in the subscale “Avoidant coping” have been discarded, as they represent undesirable coping strategies. Same questions for all surveys.  Example of item: “I've been looking for something good in what is happening”, response options: “I haven’t been doing this at all”, “A little bit”, “A medium amount”, “I’ve been doing this a lot” |
| Self-esteem | 6 items from Rosenberg’s self-esteem scale each scored 1-4 (6-24 total score). Same questions for all survey.  Example of item: “I think I have a number of good qualities”, response options: “Strongly agree”, “Agree”, “Disagree”, “Strongly disagree” |
| Sense of coherence | 4 items from the adapted version of Antonovsky's Orientation to Life Questionnaire, short form (SOC-13) fitted for adolescents each scored from 1-5 (sum score on 4-20) (25, 26). One question has a slightly different wording in the survey in 2004 compared to the rest of the waves.  Example of item: “How often do you have the feeling that there’s little meaning in the things you do in your daily life?”, response options: “Very often”, “Often”, “Sometimes”, “Rarely”, “Never” |
| **Health factors** | |
| Stress | 4 items from the Perceived stress scale (PSS) scored 0-4 (total score 0-16) (27). Adolescent version in 2004-2010, adult version in 2017-2021.  Example of item: “In the last month, how often have you felt that you were unable to control the important things in your life?”, response options: “Never”, “Almost never”, “Sometimes”, “Fairly often”, “Very often” |
| Self-rated health | 1 item from SF-36 scored 1-5 (28). Same question for all surveys.  Item: “In general, would you say your health is:”, response options: “Excellent”, “Very good”, “Good”, “Fair”, “Poor” |
| Psychosomatic symptoms | 5 items from the Hopkins Symptom Checklist 90 each scored 1-4 (5-20 total score) (29). Same questions for all surveys.  Example of item: “How much have you been bothered by: a) Headaches”, response options: “Not at all”, “A little”, “Moderately”, “Quite a bit”, “Extremely” |
| **Lifestyle factors** | |
| Physical activity | 1 item from Youth Risk Behavior Survey 2003 with a score of 1-6 (30). In the surveys in 2017-2021, the most extreme category of 7+ hours is divided in two categories of 7-10 hours and 11+. These was categorized together to ensure same scoring in all survey waves.  Item: “In a normal week, how many hours do you exercise or participate in physical that make you sweat and breathe hard, such as basketball, soccer, running, swimming laps, fast bicycling, fast dancing, or similar aerobic activities?”, response options: “None”, “About ½ hour”, “1 hour”, “2-3 hours”, “4-6 hours”, “7 or more hours” |
| Smoking | 1 item from Youth Risk Behavior Survey 2003 scored 1-4 (30). In the surveys 2007-2021 the answer “No” is dived into “No, never smoked” and “No, but smoked previously”. These was handled as “No” to secure same scoring of all surveys.  Item: “Do you smoke?”, response options: “No, I don’t smoke”, “Yes, but less often than each week”, “Yes, not daily, bit at least once a week”, “Yes, daily” |
| BMI | Based on self-reported height and weight. BMI = weight/height^2.  Items used:  What is your height in cm? _______ cm  What is your weight in kg? _______ kg |
| **Social factors** | |
| Bully | 1 item from the Health Behavior in School Children study (HBSC) with a score of 1-5. In 2004, 2007 and 2010 the question concerns bullying at school/education (31). In 2007, 2017 and 2021 the question concern bullying at work. In 2007 the answer with the highest score of either bulling at work or education was used.  Example of item: “How much have you been bullied at school during the last six months?”, response options: “I have not been bullied”, “Once or twice”, “Sometimes”, “Once a week”, “Several times a week” |
