# Supplementary material for: Estimating the associations between personal, health, lifestyle, and social factors on depressive symptoms from adolescence to adulthood: a fixed effect approach using panel data
Source: Eur J Public Health. 2026 Aug 4;36(5):ckag122. doi: 10.1093/eurpub/ckag122 (PMC13437016; doi:10.1093/eurpub/ckag122)
Supplement: Supplementary file 1 [file ckag122_supplementary_data.zip › ejph-2025-05-om-0371-File008.docx]

**Supplementary text 1: Covariates**

Data on sex and country of origin was obtained from the Population Register (31). Country of origin was defined as born in Denmark or outside Denmark.

Mental disorder diagnoses were obtained from the Danish National Patient Register from 1995 and onwards and thereby from the age of 6 in the present population born in 1989 (32, 33). Diagnoses were obtained from both psychiatric and somatic units and both primary and secondary diagnoses were included. Diagnoses were defined by the ICD-10 codes F10-F69 and F80-F99, excluding organic disorders (F00-F09) and intellectual disabilities (F70-79) given that these disorders either have onset at old ages or are congenital. The mental disorder diagnoses were described as "any mental disorder diagnosis", a dichotomized measure of present/not present for each age of interest (15-32) and within the following categories: child (age 6-12), adolescent (age 13-17), adult (age 18-32), and throughout the study period.

Medication use was defined as prescriptions for psychotropic medication using Anatomical Therapeutic Chemical codes N05A (minus N05AN), N05AN, N05B, N05C, N06A, N06B, N06C (minus N06AX01 and N06AX02), N07BB and N07BC), obtained from the Danish National Prescription Register (34). The adolescents’ medication use was described as present or not present for each age of interest (age 15-32) and within the following categories: young child (age 0-4), older child (age 5-12), adolescent (age 13-17), adult (age 18-32), and throughout the study period (age 0-32).

Equalized family income from 2004 to 2021 was obtained from the Register of Family Income. Three income groups were defined by the OECD definition of income quintile share ratio or the S60/S20 ratio: the low-income group was the families with the 20% lowest income, the high-income group was the families with the 20% highest income and the middle-income group was the families with incomes in between (35). The legal parents' highest educational level in 2004 was used as measure of parental educational level. Data on both legal parents’ educational level was obtained from Register of the Highest Completed Education (36). The highest educational levels of the parents’ were categorized into three groups: the low educational level group contained individuals with completion of up to secondary school (International Standard Classification of Education (ISCED): 0-2), the middle educational level group contained individuals with upper secondary school, vocational education, or short-cycle tertiary education (ISCED: 3-5) and the high educational level group contain individuals with a bachelor's degree or higher (ISCED: 6-8).

From the first survey wave in 2004, two measure of SES was used; subjective social status (SSS) in School and SSS in society. SSS in school and SSS in society were measured at age 15 with the MacArthur Scale of Subjective Social Status – youth version (MacArthur scale). The adolescents were asked to place themselves on a 10-step ladder representing the social hierarchy in their class (SSS in school) and to place their family on a 10-step social ladder representing the society (SSS in society) (37). Three groups of SSS were defined: low (steps 1-4), middle (steps 5-8) and high (steps 9-10).
