# Supplementary material for: Estimating the associations between personal, health, lifestyle, and social factors on depressive symptoms from adolescence to adulthood: a fixed effect approach using panel data
Source: Eur J Public Health. 2026 Aug 4;36(5):ckag122. doi: 10.1093/eurpub/ckag122 (PMC13437016; doi:10.1093/eurpub/ckag122)
Supplement: Supplementary file 1 [file ckag122_supplementary_data.zip › ejph-2025-05-om-0371-File010.docx]

| Supplementary Table 2: Dropout analyses of characteristics of analytical sample and excluded at baseline (2004) | | | |
| --- | --- | --- | --- |
|  | **Excluded** | **Analytical sample**  **(without weights)** | **Analytical sample**  **(with weights)** |
| Covariates N(%) | (N=1523) | (N=2157) | (N=2157) |
| Sex  Male  Female  Missing | 980 (64.35%)  543 (35.65%)  0 (0% of total) | p<0.05  923 (42.79%)  1234 (57.21%)  0 (0% of total) | p<0.05  1122 (52.00%)  1035 (48.00%)  0 (0% of total) |
| Country of origin  Denmark  Other  Missing | 1368 (89.82%)  155 (10.18%)  0 (0% of total) | p<0.05  2092 (96.99%)  65 (3.01%)  0 (0% of total) | p<0.05  2035 (94.36%)  122 (5.64%)  0 (0% of total) |
| Educational level  Long  Middle  Short  Missing | 58 (4.03%)  1072 (74.50%)  309 (21.47%)  84 (6% of total) | p<0.05  157 (7.31%)  1756 (81.75%)  235 (10.94%)  9 (0% of total) | p<0.05  128 (5.95%)  1666 (77.57%)  354 (16.48%)  9 (0% of total) |
| Household income  High  Middle  Low  Missing | 259 (17.01%)  855 (56.14%)  409 (26.85%)  0 (0% of total) | p<0.05  476 (22.07%)  1353 (62.73%)  328 (15.21%)  0 (0% of total) | p<0.05  413 (19.16%)  1332 (61.77%)  411 (19.07%)  0 (0% of total) |
| SSS in society  Long  Middle  Short  Missing | 46 (4.91%)  486 (51.92%)  404 (43.16%)  587 (39% of total) | p=0.44  581 (4.02%)  1032 (51.22%)  902 (44.76%)  142 (7% of total) | p=0.98  95 (4.74%)  1044 (51.82%)  875 (43.44%)  142 (7% of total) |
| SSS in school  Long  Middle  Short  Missing | 14 (1.48%)  603 (63.61%)  331 (34.92%)  575 (38% of total) | p=0.376  34 (1.68%)  1334 (65.94%)  655 (32.38%)  134 (6% of total) | p=0.07  41 (2.01%)  1359 (67.16%)  624 (30.83%)  134 (6% of total) |
| Mental Disorder |  |  |  |
| Older child (5-12 years)  None as child | 63 (4.14%)  1460 (95.86%) | 44 (2.04%) p<0.05  2113 (97.96%) | 64 (2.96%) p=0.09  2093 (97.04%) |
| Adolescent (13-17 years)  None as adolescent | 99 (6.50%)  1424 (93.50%) | 79 (3.66%) p<0.05  2078 (96.34%) | 106 (4.92%) p=0.08  2051 (95.08%) |
| Adult (18-32 years)  None as adult | 323 (21.21%)  1200 (79.79%) | 310 (14.37%) p<0.05  1847 (85.63%) | 373 (17.30%) p<0.05  1784 (82.70%) |
| Study period (5-32 years)  None in study period | 397 (26.07%)  1126 (73.93%) | 361 (16.74%) p<0.05  1796 (83.26%) | 451 (20.93%) p<0.05  1706 (79.07%) |
| Missing | 0 (0% of total) | 0 (0% of total) | 0 (0% of total) |
| Medication use |  |  |  |
| Older child (5-12 years)  None as child | 26 (1.71%)  1497 (98.39%) | 13 (0.60%) p<0.05  2144 (99.40%) | 22 (1.02%) p=0.13  2135 (98.98%) |
| Adolescent (13-17 years)  None as adolescent | 65 (4.27%)  1458 (95.61%) | 53 (2.46%) p<0.05  2104 (97.54%) | 66 (3.04%) p=0.07  2091 (96.96%) |
| Adult (18-32 years)  None as adult | 520 (34.14%)  1003 (65.86%) | 500 (23.18%) p<0.05  1657 (76.82%) | 601 (27.85%) p<0.05  1556 (72.15%) |
| Study period (5-32 years) None in study period | 575 (37.75%)  948 (62.25%) | 559 (25.92%) p<0.05  1598 (74.08%) | 667 (30.92%) p<0.05  1490 (69.08%) |
| Missing | 0 (0% of total) | 0 (0% of total) | 0 (0% of total) |
