# Supplementary material for: Estimating the associations between personal, health, lifestyle, and social factors on depressive symptoms from adolescence to adulthood: a fixed effect approach using panel data
Source: Eur J Public Health. 2026 Aug 4;36(5):ckag122. doi: 10.1093/eurpub/ckag122 (PMC13437016; doi:10.1093/eurpub/ckag122)
Supplement: Supplementary file 1 [file ckag122_supplementary_data.zip › ejph-2025-05-om-0371-File011.docx]

| **Supplementary Table 3**: Number and prevalence of with-in person change in explanatory variables between age point | | | | |
| --- | --- | --- | --- | --- |
|  | **Changed N(%)** | | | |
|  | Age 15-18 | Age 18-21 | Age 21-28 | Age 28-32 |
| **Personal factors** |  |  |  |  |
| **Coping** | 2023 (94%) | 2039 (95%) | 1907 (88%) | 1887 (87%) |
| **Self-esteem** | 1825 (85%) | 1743 (81%) | 1768 (82%) | 1698 (79%) |
| **Sense of coherence** | 1806 (84%) | 1777 (82%) | 1790 (83%) | 1750 (81%) |
| **Health factors** |  |  |  |  |
| **Stress** | 1858 (86%) | 1860 (86%) | 1865 (86%) | 1847 (86%) |
| **Psychosomatic symptoms** | 1763 (82%) | 1735 (80%) | 1782 (83%) | 1663 (77%) |
| **Self-rated health**  Excellent  Very good  Good  Fair  Poor | 1235 (57%) | 1217 (56%) | 1244 (58%) | 1122 (52%) |
| **Lifestyle factors** |  |  |  |  |
| **Physical activity**  7+ hours  4-6 hours  2-3 hours  1 hour  1/2 hour  None | 1417 (66%) | 1476 (68%) | 1535 (71%) | 1508 (70%) |
| **Smoking**  No  Rarely  Weekly  Daily | 565 (26%) | 462 (21%) | 461 (21%) | 421 (20%) |
| **BMI** | 2146 (99%) | 2119 (98%) | 2111 (98%) | 2088 (97%) |
| **Social factors** |  |  |  |  |
| **Bullying**  Never  Once or twice  Sometimes  Weekly  Several times a week | 685 (32%) | 424 (20%) | 301 (14%) | 308 (14%) |
