# Supplementary material for: Estimating the associations between personal, health, lifestyle, and social factors on depressive symptoms from adolescence to adulthood: a fixed effect approach using panel data
Source: Eur J Public Health. 2026 Aug 4;36(5):ckag122. doi: 10.1093/eurpub/ckag122 (PMC13437016; doi:10.1093/eurpub/ckag122)
Supplement: Supplementary file 1 [file ckag122_supplementary_data.zip › ejph-2025-05-om-0371-File012.docx]

| Supplementary Table 4: P-values of Hausmans test on FE and POLS | | | | |
| --- | --- | --- | --- | --- |
|  | 2004-2007 | 2007-2010 | 2010-2017 | 2017-2021 |
| p-values | 0.295 | 0.063 | 0.099 | 0.117 |
