# Supplementary material for: Estimating the associations between personal, health, lifestyle, and social factors on depressive symptoms from adolescence to adulthood: a fixed effect approach using panel data
Source: Eur J Public Health. 2026 Aug 4;36(5):ckag122. doi: 10.1093/eurpub/ckag122 (PMC13437016; doi:10.1093/eurpub/ckag122)
Supplement: Supplementary file 1 [file ckag122_supplementary_data.zip › ejph-2025-05-om-0371-File013.docx]

| **Supplementary Table 5:** Results from Fixed Effect (FE) regressions and dominance analyses of relative contribution to explained variance | | | | | | | | |
| --- | --- | --- | --- | --- | --- | --- | --- | --- |
|  | **FE effects regression coefficients (95% CI)** | | | | **Relative contribution to explained variance (%)** | | | |
|  | **Age 15-18** | **Age 18-21** | **Age 21-28** | **Age 28-32** | **Age 15-18** | **Age 18-21** | **Age 21-28** | **Age 28-32** |
| **Explained total variance** | 0.29 | 0.25 | 0.27 | 0.29 |  |  |  |  |
| **Personal factors** |  |  |  |  |  |  |  |  |
| **Coping** | 0.00 (-0.02-0.02) | -0.00 (-0.02-0.02) | 0.02 (-0.02-0.06) | 0.05 (0.00-0.09) | 0.05 | 0.09 | 4.79 | 6.08 |
| **Self-esteem** | 0.06 (0.01-0.11) | 0.14 (0.09-0.18) | 0.10 (0.05-0.15) | 0.11 (0.05-0.17) | 8.58 | 21.14 | 17.8 | 17.01 |
| **Sense of coherence** | 0.16 (0.10-0.21) | 0.16 (0.11-0.21) | 0.18 (0.12-0.24) | 0.15 (0.08-0.22) | 16.81 | 21.22 | 24.51 | 22 |
| **Health factors** |  |  |  |  |  |  |  |  |
| **Stress** | 0.25 (0.19-0.30) | 0.22 (0.17-0.27) | 0.19 (0.13-0.24) | 0.17 (0.11-0.22) | 31.33 | 36.13 | 33.23 | 30.94 |
| **Psychosomatic symptoms** | 0.11 (0.07-0.15) | 0.06 (0.01-0.10) | 0.05 (0.01-0.10) | 0.08 (0.03-0.12) | 8.88 | 3.94 | 4.92 | 5.48 |
| **Self-rated health**  Excellent (ref)  Very good  Good  Fair  Poor | 0.00 ( .- .)  -0.15 (-0.38-0.08)  0.12 (-0.18-0.42)  0.57 (-0.04-1.17)  1.32 (0.14-2.50) | 0.00 ( .- .)  0.09 (-0.13-0.30)  0.28 (-0.02-0.58)  0.48 (-0.13-1.09)  0.30 (-0.82-1.43) | 0.00 ( .- .)  0.21 (-0.02-0.44)  0.49 (0.14-0.84)  0.54 (-0.02-1.10)  0.95 (-0.34-2.23) | 0.00 ( .- .)  0.25 (-0.02-0.51)  0.52 (0.15-0.89)  0.85 (0.25-1.44)  1.24 (0.10-2.38) | 8.88 | 5.71 | 8.31 | 11.31 |
| **Lifestyle factors** |  |  |  |  |  |  |  |  |
| **Physical activity**  7+ hours (ref)  4-6 hours  2-3 hours  1 hour  1/2 hour  None | 0.00 ( .- .)  0.14 (-0.12-0.41)  0.19 (-0.12-0.50)  -0.03 (-0.41-0.36)  -0.11 (-0.68-0.45)  0.12 (-0.51-0.76) | 0.00 ( .- .)  -0.04 (-0.32-0.25)  0.05 (-0.26-0.36)  0.07 (-0.33-0.48)  0.28 (-0.21-0.76)  0.23 (-0.29-0.75) | 0.00 ( .- .)  0.15 (-0.17-0.47)  0.20 (-0.14-0.53)  0.00 (-0.39-0.40)  0.19 (-0.28-0.67)  0.06 (-0.51-0.63) | 0.00 ( .- .)  0.11 (-0.23-0.45)  0.15 (-0.21-0.51)  -0.03 (-0.46-0.40)  0.08 (-0.39-0.55)  -0.10 (-0.64-0.43) | 1.1 | 1.22 | 1.59 | 1.62 |
| **Smoking**  No (ref)  Rarely  Weekly  Daily | 0.00 ( .- .)  0.07 (-0.33-0.46)  -0.10 (-0.60-0.41)  -0.22 (-0.65-0.21) | 0.00 ( .- .)  -0.09 (-0.49-0.31)  -0.39 (-0.87-0.08)  -0.22 (-0.70-0.26) | 0.00 ( .- .)  0.27 (-0.14-0.68)  -0.17 (-0.75-0.41)  0.11 (-0.30-0.51) | 0.00 ( .- .)  0.14 (-0.30-0.57)  -0.10 (-0.87-0.66)  0.03 (-0.45-0.51) | 0.37 | 0.68 | 1.42 | 0.71 |
| **BMI** | 0.01 (-0.04-0.07) | 0.02 (-0.04-0.07) | -0.03 (-0.07-0.01) | 0.05 (-0.01-0.10) | 0.22 | 0.16 | 0.38 | 1.8 |
| **Social factors** |  |  |  |  |  |  |  |  |
| **Bullied**  Never (ref)  Once or twice  Sometimes  Weekly  Several times a week | 0.00 ( .- .)  0.21 (-0.10-0.52)  0.67 (0.16-1.18)  1.32 (0.33-2.32)  0.19 (-1.16-1.54) | 0.00 ( .- .)  0.22 (-0.17-0.60)  0.35 (-0.60-1.29)  -0.20 (-1.76-1.35)  -1.00 (-3.87-1.88) | 0.00 ( .- .)  0.05 (-0.49-0.60)  -0.21 (-1.35-0.93)  -0.25 (-1.65-1.15)  -0.27 (-2.79-2.25) | 0.00 ( .- .)  0.27 (-0.30-0.83)  0.25 (-0.53-1.04)  0.48 (-0.47-1.43)  0.00 ( .- .) | 6.03 | 2.18 | 1.53 | 2.72 |
