# Supplementary material for: Estimating the associations between personal, health, lifestyle, and social factors on depressive symptoms from adolescence to adulthood: a fixed effect approach using panel data
Source: Eur J Public Health. 2026 Aug 4;36(5):ckag122. doi: 10.1093/eurpub/ckag122 (PMC13437016; doi:10.1093/eurpub/ckag122)
Supplement: Supplementary file 1 [file ckag122_supplementary_data.zip › ejph-2025-05-om-0371-File014.docx]

**Supplementary text 2:** Additional references

41. Luchman JN. Determining relative importance in Stata using dominance analysis: domin and domme. The Stata journal. 2021;21(2):510-38.

42. Scheid TL, Wright ER. A handbook for the study of mental health : social contexts, theories, and systems. Third edition. ed. Cambridge: Cambridge University Press; 2017.

43. Biering K, Schougaard LMV, Steen PB, Dørken EV, Glasscock D, Sørensen CLB. Translation, cross-cultural adaptation, and reliability of the Center for Epidemiological Studies Depression Scale for Children (CES-DC) in Danish adolescents (under review). BMC Pediatrics. 2025
